# Fiber-Reinforced Polymer-Confined Non-Circular Columns with Shape Modification: A Comprehensive Review

**DOI:** 10.3390/polym14030564

**Published:** 2022-01-30

**Authors:** Chunbao He, Jun-Jie Zeng

**Affiliations:** 1 College of Water Conservancy and Civil Engineering, South China Agricultural University, Guangzhou 510642, China; 2 School of Civil and Transportation Engineering, Guangdong University of Technology, Guangzhou 510006, China; 3 Department of Civil and Environmental Engineering, University of Macao, Macao 999078, China

**Keywords:** reinforced concrete (RC) column, fiber-reinforced polymer (FRP), section modification, confinement, section curvilinearization (SC), stress–strain model

## Abstract

The implementation of shape modification (SM) to reinforced concrete (RC) columns has been demonstrated to be effective when enhancing the effectiveness of the fiber-reinforced polymer (FRP) confinement of the columns, particularly for non-circular columns. The SM approach generally includes modifying a square section into a circular one, modifying a rectangular section into an elliptical/oval one and modifying a square/rectangular section into a curvilinearized square/rectangular section. In this paper, a state-of-the-art review of studies on FRP-confined non-circular columns with SM is conducted. The effects of key parameters on the effectiveness of FRP confinement are discussed, and different methods for the implementation of SM in real applications are briefly introduced. The findings of the review further confirm the effectiveness of the SM approach, and the test results demonstrate the effectiveness and advantages of section curvilinearization with a limited increase in cross-sectional area. Additionally, existing theoretical models for FRP-confined concrete in columns with SM are summarized. Further research opportunities associated with FRP-confined non-circular columns with SM are identified.

## 1. Introduction

**Figure 1:**
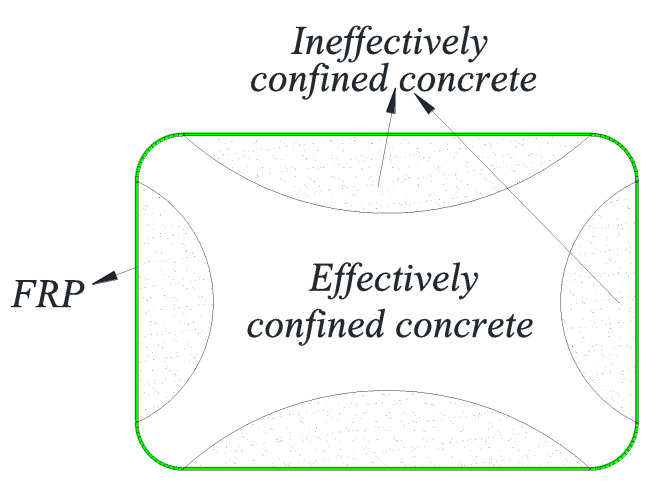
Effectively and ineffectively confined concrete in rectangular columns.

Fiber-reinforced polymer (FRP) composites have been widely used for strengthening engineering structures [1,2,3,4,5,6,7,8,9,10,11]. FRP jacketing in particular is a widely-accepted technique for strengthening reinforced concrete (RC) columns [12,13,14,15,16,17,18,19]. Based on extensive experimental and theoretical studies, FRP jacketing has been demonstrated to be an effective strengthening technique for both circular and non-circular columns [20,21,22,23,24,25,26]. RC columns with FRP wrapping exhibit excellent load carrying and deformation capacities because the dilation of concrete under compression is well confined by the FRP wrap. However, while the confinement efficiency of FRP for circular columns is satisfactory, FRP wrapping of non-circular columns (including square columns as a special case) is much less effective than the former due to the presence of sharp corners and non-uniform confinement (Figure 1) [27,28,29,30,31]. Although corner rounding has been proposed for non-circular columns before FRP wrapping [32,33,34,35], its effectiveness is still limited because the corner radius is limited due to the existence of internal steel reinforcement in RC columns [15,36,37].

**Figure 2:**
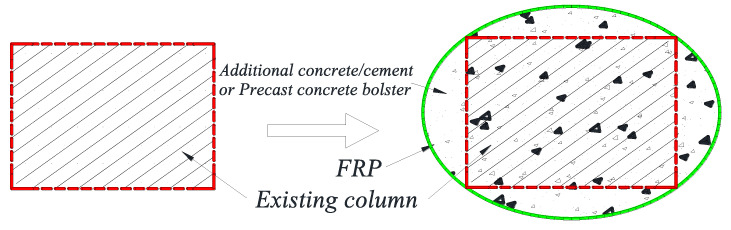
Section modification for square/rectangular columns before FRP jacketing.

Two decades ago, researchers proposed the shape modification (SM) technique to facilitate the enhancement of the effectiveness of the FRP wrapping strengthening technique for RC columns. SM generally refers to implementing an appropriate cross-sectional SM (e.g., modifying a rectangular section into an oval or elliptical section) prior to FRP wrapping [38,39] by attaching precast concrete bolsters or casting additional concrete/cement in a stay-in-place formwork with a desired sectional shape (Figure 2). Seible and Priestley [38] adopted an elliptical steel tube as the external confining tube to strengthen rectangular RC columns, and subsequently Priestley and Seible [40] adopted the same SM technology with respect to rectangular RC columns before FRP wrapping. Based on this concept, SM of a rectangular section into an oval or elliptical section or a square section into a circular section (referred to as section circularization or section ellipticalization) prior to FRP wrapping has been studied by many researchers [41,42,43,44,45,46,47,48,49,50,51,52]. It should be known that in this review, corner rounding is not regarded as a process of SM. However, the primarily drawback of SM is that it introduces a substantial increase in the cross-sectional area of the columns and thus the dead weight, which is not good in terms of the seismic performance of the structural system. Also, an increase in the column’s cross-sectional area means a reduction in the usable floor area. 

**Figure 3:**
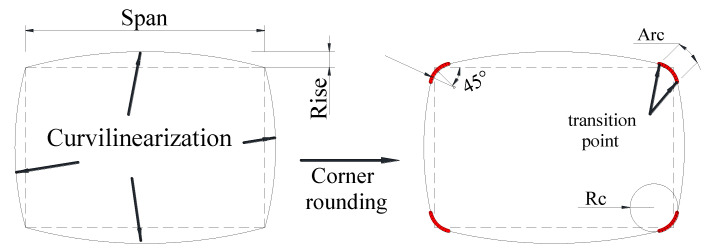
SC for square/rectangular columns before FRP jacketing.

**Figure 4:**
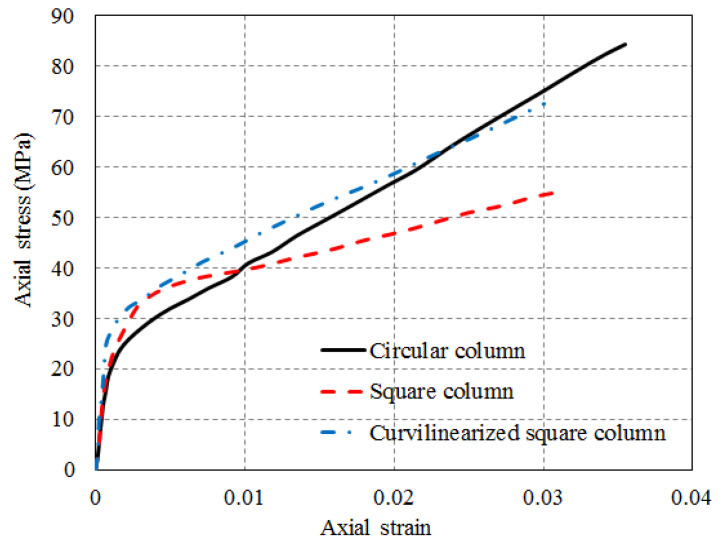
Stress–strain of FRP-confined concrete in circular, square and curvilinearized columns [56].

To this end, the section curvilinearization (SC) approach (regarded as a form of SM technique in this paper) has been proposed and investigated by some researchers [53,54,55,56]. In this approach, the flat sides of a rectangular column are transformed into only slightly curved sides before FRP wrapping (Figure 3). Therefore, the difference between the SC approach and the conventional SM approach is that the SC approach only leads to a slight increase in the column cross-sectional area, which is favorable for designers and building users. The resulting column is referred to as a curvilinearized rectangular column (CRC), and FRP confinement effectiveness has been found to be substantially enhanced [56,57,58,59,60] even though the cross-sectional area of the column is only marginally increased (Figure 4). This is because the jacket bending action is converted to a membrane action in confining the dilating concrete, and the tensile capacity of the fibers in the FRP jacket is sufficiently mobilized in a CRC. In the practical method, the nominal rise-to-span (r/s) ratio of the curvilinearized section is defined to be the r/s ratio of the curved molds in step (1) (Figure 3) rather than the actual r/s ratio of the column section after curvilinearization (Figure 3). Studies on the axial and eccentric compressive behavior of FRP-confined CRCs have been conducted [54,55,56,57,58,59,60], and the efficiency of the SC approach has been demonstrated.

On the theoretical side, Lai et al. [56] proposed an axial stress–axial strain model (simply named a “stress–strain model” hereafter) for FRP-confined concrete in CSCs based on their own test data. Zhu [57] carried out a finite element (FE) study on FRP-confined curvilinearized square columns (CSCs) under axial compression and proposed a stress–strain model for FRP-confined concrete in CSCs based on their FE results as well as their test results (Zhu et al. [59]). The models of Lai et al. [56] and Zhu [57], however, do not include either the r/s ratio or the corner radius ratio as parameters and are only applicable to FRP-confined CSCs. Another stress–strain model for FRP-confined concrete in CRCs is given in the Chinese national standard for the structural use of FRP composites in construction [35] (referred to as the Chinese national standard). That model was established on the basis of the test results of small-scale FRP-confined CSCs from Lai et al. [56]. Its applicability to FRP-confined concrete in large-scale CRCs had been investigated by Zhu et al. [59] and Zeng et al. [60], and a new version of the stress–strain model for concrete in CRCs has been introduced based on test results from Zeng et al. [60].

In this article, a comprehensive review of studies on FRP-confined non-circular columns with SM is conducted. The effects of key parameters on the effectiveness of FRP strengthened concrete columns are discussed, and the implementation of SM in real applications is briefly introduced. The findings of the review further confirm the effectiveness of the SM approach. Additionally, existing theoretical models for FRP-confined concrete with SM are summarized. Further research opportunities associated with FRP-confined non-circular columns with SM are identified. It should be noted that studies on the effect of the corner rounding method are not reviewed in this paper, as the cross-sectional shape in this approach is generally unchanged.

## 2. Implementation of SM before FRP Wrapping

### 2.1. Implementation of SM before FRP Wrapping for Strengthening/Repairing Existing RC Columns

**Figure 5:**
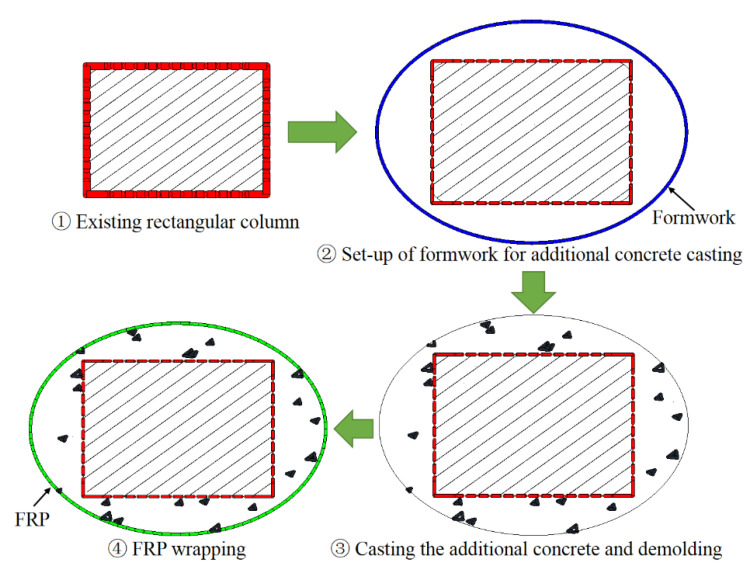
Section circularization/ellipticalization based on formwork and fresh concrete/cement mortar.

**Figure 6:**
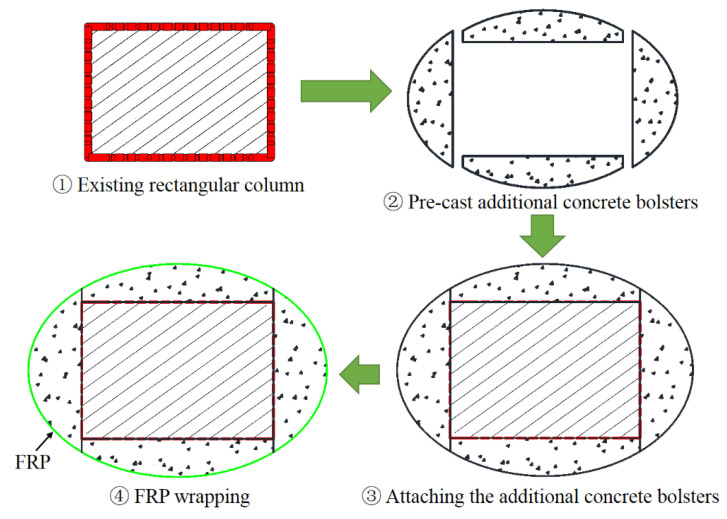
Section circularization/ellipticalization based on pre-cast additional concrete bolsters.

**Figure 7:**
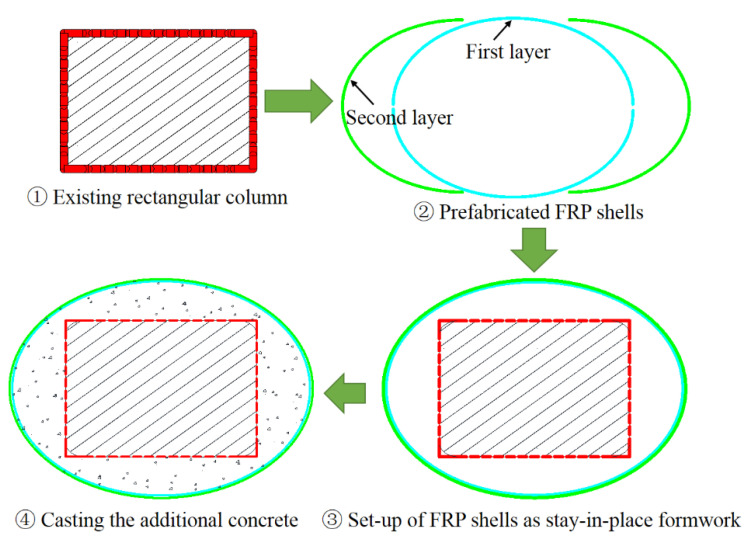
Section circularization/ellipticalization based on stay-in-place FRP shells.

**Figure 8:**
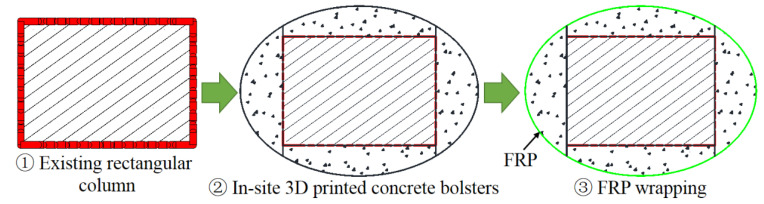
Section circularization/ellipticalization based on 3D in-site printed concrete/cement mortar bolsters.

The approaches of section circularization and section ellipticalization can be generally achieved by the following means: (i) casting concrete/cement mortar into the gap between the existing square/rectangular column and the circular/elliptical formwork with required cross-sectional dimensions before FRP wrapping (Figure 5); (ii) attaching pre-cast concrete bolsters to the four sides of a square/rectangular column before FRP wrapping (Figure 6); (iii) casting concrete/cement mortar into the gap between the existing square/rectangular column and the circular/elliptical FRP prefabricated shells (Figure 7); (iv) SM with advanced construction techniques such as the 3D on-site printing of the additional concrete/cement mortar bolsters for existing square/rectangular columns (Figure 8).

For the first approach, the required setting up of the formwork and re-molding are labor-consuming, and the formwork needs to be prepared in separated halves so that they can be set-up in the required position for real columns as the in-service columns are well connected to other structural members. In the second approach, the process of pre-casting concrete bolsters is time and labor-consuming because additional formworks are needed for casting the concrete bolsters and it takes some time for the pre-cast concrete bolsters to cure before FRP wrapping. Both the first and second approaches require the installation of FRP wrap on the column after the section modification process. The third method involves pre-fabricated FRP shells, meaning that additional formworks and FRP wrap installation after section modification are unnecessary, which is cost-effective and labor-saving. However, the pre-fabricated FRP shells also need to be prepared and installed in two separated halves (breaking the FRP shell into two halves) so that they are applicable to columns in practice. This means that the confinement effectiveness of the FRP shell is reduced due to the non-continuous fibers in the hoop direction of the separated halves of the FRP shell. The first three approaches have been realized in practice, while the last approach represents a new way to implement the SM because only a printing machine is required, which saves time and labor. The mentioned approaches are applicable in both experimental works and in real applications with respect to the strengthening/repairing of RC columns. However, the effects of these different SM approaches on the FRP confinement mechanism, particularly the effects of the second and the third approaches, remain unclear.

The SC approach has been realized by Zeng et al. [60] in an experimental study by casting whole curvilinearized columns using wooden molds with the required shape. Similar to section circularization and ellipticalization, SC needs to be implemented on an existing RC column in practical applications. The four approaches of section circularization and ellipticalization mentioned previously are basically applicable to SC. However, prefabricated square/rectangular FRP shells with slightly curved sides are not easy to prepare, and therefore the first approach, which involves casting additional concrete bolsters, is the most feasible approach for SC.

**Figure 9:**
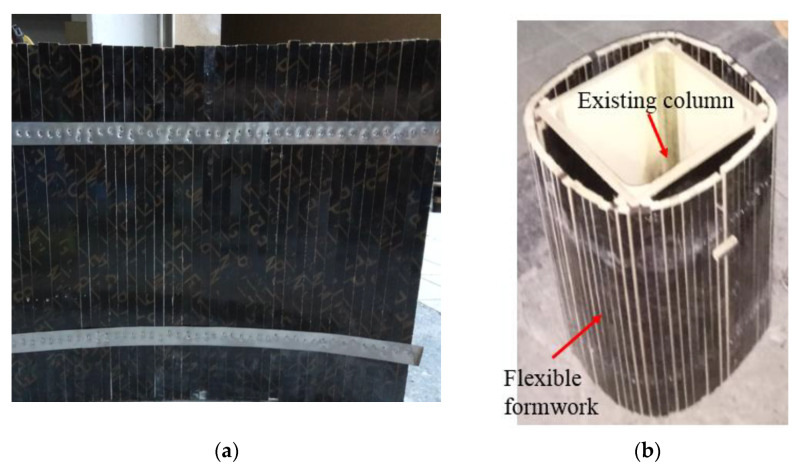
Novel flexible formwork system for SC [61]. (**a**) Linking parallel wooden bars with steel strips. (**b**) Wrapping the flexible formwork around an existing column.

In Zeng 2017 [61], a novel formwork was developed for SC. This novel formwork system consists of a series of parallel wooden bars linked together by two steel strips (Figure 9). The formwork is to be wrapped around an existing column; a number of positioning wooden blocks between the existing column and the formwork help to control the shape of the formwork to achieve the desired sectional shape (see Figure 9). In addition, due to the flexibility of the formwork, the pressure from the filled wet concrete can automatically ensure the desired sectional shape for the given circumferential length of the formwork (i.e., the number of wooden bars), which can easily be adjusted to suit different column sizes.

**Figure 10:**
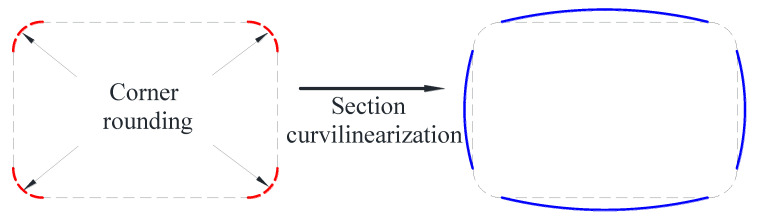
SC procedures in practical applications.

It should be noted that the corners of the strengthened column are rounded after SC (see Figure 3). However, the corners can be rounded before SC in practical applications (Zhu et al. [59]) so that the span of the section is reduced, and the rise of the curved side is subsequently reduced, leading to a reduced increase in the cross-sectional area of the strengthened column. Therefore, SC can be implemented using two methods: (i) rounding the corners after SC (Figure 3); (ii) rounding the corners before SC (Figure 10). Due to the rounded corners in the second method, the actual rise of the column side, which is defined as the highest point of the curve to the original flat side, is smaller using this method than it is in the former method (see Figure 10). Nonetheless, the two methods lead to the same curvature for the curved sides if the same nominal r/s ratio of the flat sides is used. For example, for a specimen with an r/s ratio of 1/10, the actual rise of the flat sides using the practical method is 50% lower than the nominal rise of 15 mm, which leads to a 15% smaller cross-sectional area. For a comparison of the effectiveness of the two forming methods, Zhu et al. [59] conducted a finite element analysis, and it was found that the predicted stress–strain curves of concrete in CRCs using the two different SC methods are almost identical. This also suggests that the curvature of the flat side and the corner radius ratio are the two key parameters affecting the behavior of concrete in FRP-confined CSCs, as will be reviewed in the subsequent section.

In practical applications, the strength of the new concrete filled in the gaps between the curved molds and the existing column may be higher than that of the concrete of the existing column. A finite element study presented in Zhu et al. [59] found that the average axial stress–axial strain curve for a curvilinearized column filled with concrete of a higher compressive strengths is approximately identical to the curve of a corresponding column filled with concrete of the same strength. It is believed that in practical applications, the influence of the concrete strength difference is very small as long as the difference in the compressive strengths of the old concrete and the new concrete is not so large. It should be mentioned that the Chinese standard (GB 50608 [35]) recommends that the cross-section area of the original section before SM be used in the calculation of axial stress for a curvilinearized column to ensure a conservative approach.

### 2.2. New Structural Members with SC and FRP Confinement

**Figure 11:**
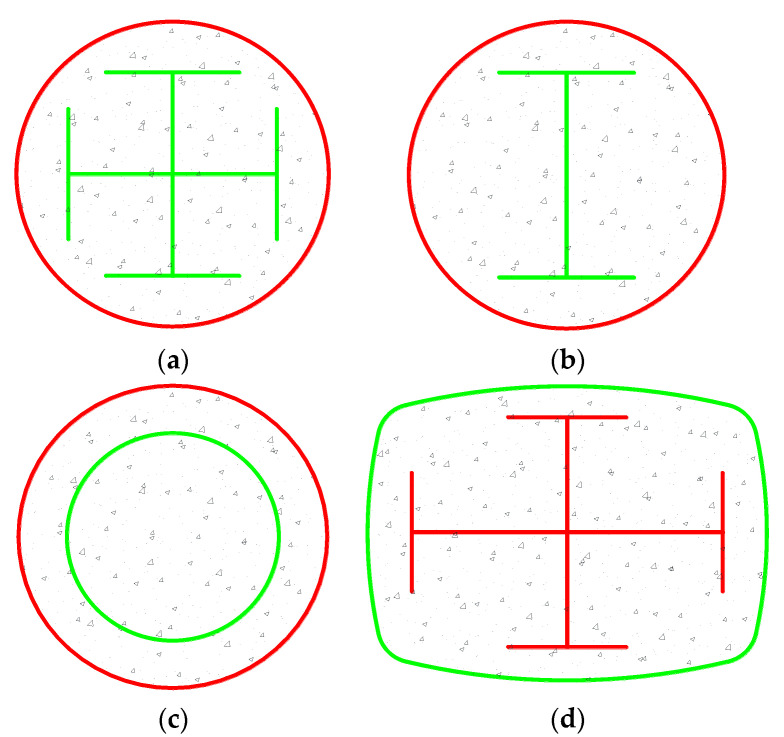
Circular FCSRCs with different shapes of steel sections and a curvilinearized rectangular FCSRC. (**a**) Circular FCSRC with a cruciform steel section. (**b**) Circular FCSRC with an I steel section. (**c**) Circular FCSRC with a circular steel tube. (**d**) Curvilinearized rectangular FCSRC.

The SC method can be applied to composite structural members in which the efficiency of FRP confinement is critical. FRP confining tubes have been employed to enhance the compressive behavior of steel reinforced concrete columns with a cruciform, I or circular steel sections (Figure 11a–c), and the resulting columns are referred to as FRP-confined steel reinforced concrete columns (FCSRCs) [62,63,64,65,66,67,68]. However, confinement from FRP may not be satisfactory in a square/rectangular FCSRC due to the non-uniform distribution of the confining stresses. To this end, novel forms FCSRCs with curvilinearized square/rectangular sections are proposed in this study (see Figure 11d). The performance and confinement mechanism in curvilinearized square/rectangular FCSRCs remain to be understood.

Engineered cementitious composites (ECCs), also known as ultra-high toughness cementitious composites (UHTCCs), are a class of high-performance fiber-reinforced cementitious composites (HPFRCCs). Owing to the addition of fibers, ECCs can exhibit a large tensile strain capacity ranging from 3% to 8%, with the advantages of high fracture toughness, high tensile strength, high cracking resistance and superior durability [69,70,71,72,73,74,75,76,77,78,79,80]. As the bond strength between ECC and normal concrete is satisfactory [77], ECC can be filled in the gap between the old column and the FRP wrap to form a curvilinearized section when strengthening/repairing existing square/rectangular columns to further enhance the compression and bending load carrying capacities of the columns.

## 3. Section Circularization and Section Ellipticalization

Existing studies have demonstrated that the FRP confinement efficiency is efficient for circular columns, and although the FRP confinement efficiency for elliptical columns is less efficient, it is still satisfactory. Therefore, implementing section circularization and ellipticalization for square and rectangular columns respectively before FRP jacketing is widely accepted. Generally, the efficiency of section circularization/ellipticalization with regard to FRP confinement has been verified in existing studies [46,47,48,49,50,81,82,83,84,85].

Priestley and Seible [40] suggested modifying rectangular sections into elliptical or oval sections, and it was mentioned that rectangular columns subject to seismic loads can be effectively confined using precast circular or oval bolsters added to the plastic hinge region prior to the installation of a confining device. Alternatively, it was suggested that the corners could be rounded with a maximum corner radius before FRP wrapping. However, the confinement efficiency for columns with corner rounding is only approximately 50% of that for columns using the circularization approach. Saadatmanesh et al. [42] modified a rectangular RC column (368 × 241 mm) with concrete (with a strength of 35 MPa) into an oval shape (495 × 292 mm) by using fast curing cement and then wrapped the modified column with GFRP straps. The results demonstrated the efficiency of the approach of applying SM before FRP strengthening.

**Figure 12:**
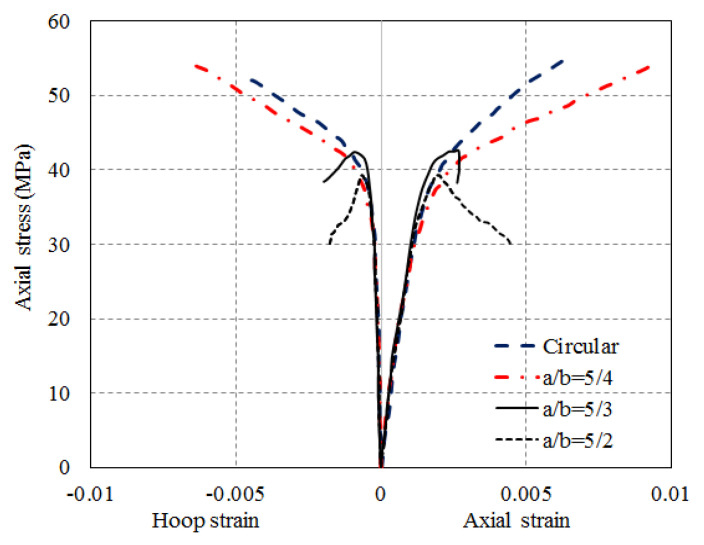
Stress–strain curves of one-layer FRP-confined concrete in elliptical columns (reproduced based on Teng and Lam [43]).

Subsequently, Teng and Lam [43] suggested that modifying a square/rectangular column into a circular/elliptical column before FRP strengthening is a viable approach. A preliminary study on the compressive behavior of CFRP-confined elliptical concrete columns was conducted to understand the compressive strength of CFRP-confined concrete in elliptical columns. Twenty specimens were tested with aspect ratios (i.e., ratios between the lengths of major and minor axes) of 1, 5/4, 5/3 and 5/2. The results reported by Teng and Lam [43] show that the axial compressive strength of FRP-confined concrete in elliptical columns is controlled by the FRP thickness and the major-to-minor axis length ratio a/b of the column section (Figure 12). The confinement effectiveness decreased with the a/b ratio but a substantial amount of strength could also be gained from FRP confinement even for strongly elliptical sections. Furthermore, the stress–strain behavior of FRP-confined concrete in elliptical columns was related to the effective confinement ratio, which is the ratio of the effective confining pressure to the strength of unconfined concrete, and the stress–strain curve exhibited a descending branch if the effective confinement ratio was equal to or less than 0.11. The results also showed that the largest strength enhancement occurred for the specimens with an aspect ratio equal to 1 (circular specimens), which achieved a 119% increase. The lowest strength enhancement occurred for specimens with the largest aspect ratio (i.e., 5/2), which achieved a 38% increase. However, Teng and Lam [43] cast the elliptical columns as a whole and only the axial compressive behavior of CFRP-confined concrete in elliptical columns was explored. The strength model for FRP-confined concrete in circular columns was modified by introducing an effective confining pressure which is equal to the product of a shape factor considering the effect of section shape and confining pressure in an equivalent circular column with the same FRP volumetric ratio.

**Figure 13:**
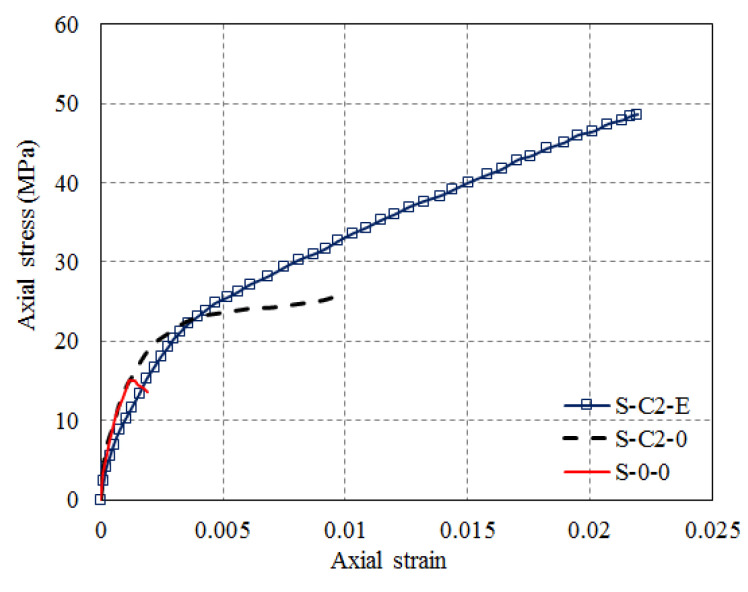
Stress–strain curves of FRP-confined concrete in square columns with section circularization (reproduced based on Yan and Pantelides [46]).

Subsequently, Yan et al. [81] conducted an experimental study into the effectiveness of SM on square and rectangular columns confined with FRP. Axial compression tests of circular and elliptical columns (with an unconfined concrete strength of 14.3 MPa) with shape circularization and the ellipticalization of square (279 × 279 mm) and rectangular (381 × 203 mm, and 457 × 152 mm) columns were carried out. SM was performed using prefabricated FRP shells with expansive cement. The expansive cement was able to expand during setting time and provide prestresses (active confinement to concrete) in the FRP shell. Test results including failure modes, stress–strain curves and the effects of SM and expansive cement were presented. It was found that a higher axial compressive strength and higher energy absorption were observed for shape-modified square and rectangular columns with post-tensioned FRP shells compared with columns confined using bonded FRP jackets with the same FRP thickness. The post-peak softening axial stress–strain behavior of concrete in FRP-confined square/rectangular columns (Specimen S-0-0) was transformed to a post-peak hardening behavior owning to the SM method (Specimen S-C2-0), and the active confinement introduced from the expansive cement (Specimen S-C2-E) enabled a better performance in confined concrete (Figure 13). Also, a higher axial compressive strength and higher energy absorption were observed for shape-modified square and rectangular columns with post-tensioned FRP shells compared with members confined using bonded FRP jackets with the same FRP thickness.

Parvin and Schroeder [82] presented a finite-element analysis of eccentrically loaded FRP confined elliptical columns. These elliptical columns were made from rectangular columns using ellipticalization. The effect of wrap configuration, including the number of layers and fiber orientation, on the performance of such columns under eccentric loading scenarios was explored. The results showed that the effectiveness of CFRP wrapping was substantially reduced for eccentrically loaded columns compared with concentrically loaded columns and that the CFRP jacket was more effective in the axial direction than the CFRP jacket in the hoop direction for eccentrically loaded columns.

Hadi et al. [47] conducted a series of tests on concentrically and eccentrically loaded FRP-confined RC square columns with section circularization, and the effect of section circularization on FRP confinement effectiveness was compared with the effect of corner rounding. Sixteen square RC columns with a cross-sectional width of 150 m and a height of 800 mm were tested. The circularization process was performed by bonding four segmental circular concrete covers and the modified columns were treated as complete circular columns. They found that both corner rounding and section circularization were effective in enhancing the compressive behavior of FRP-confined concrete in square columns. The added concrete covers effectively enhanced the load carrying capacity of the columns by increasing the cross-sectional area and increasing the effectiveness of FRP confinement. Pham et al. [48] reported an experimental study on FRP-confined square columns with segmental circular concrete covers with different concrete strengths (40 MPa, 80 MPa and 100 MPa). Square RC columns with a cross-sectional width of 150 m and a height of 800 mm were tested. The test results also demonstrated that section circularization is effective in enhancing FRP confinement efficiency. It was also verified that the concrete covers with a higher strength exhibited a higher load-carrying capacity than the concrete covers with a lower strength. 

Hadi and Tran [83] tested two RC beam-column joints with cross-sectional dimensions of 200 × 300 mm for the beams and 200 × 200 mm for the columns using the section circularization technique introduced by Hadi et al. [47]. One joint was strengthened and the other one was repaired after a serious failure caused by the applied load. The columns were only retrofitted at the point of the beam-column intersection and were tested under reversed cyclic loading. Both the original concrete joint and the circular segments had a strength of 50 MPa. The test results showed that the performances of the original columns were improved significantly after being strengthened. In addition, the circular segments worked well with the existing concrete to resist shear load. Hadi and Tran [83] then extended their experimental tests by investigating the effect of different thicknesses of FRP and found that the circularization method with an increased FRP thickness helped to relocate the failure of the beam-column connection from the joint location to any preferred location in the beam span.

Alsayed et al. [84] conducted a study on the FRP confinement efficiency of FRP-confined wall-like rectangular RC columns with section ellipticalization. The test columns had cross-sectional dimensions of 125 × 500 mm. As expected, they found that CFRP confinement increased both the strength and ductility of confined concrete in rectangular RC columns with section ellipticalization. Additionally, owning to the confinement provided by the CFRP wrap, the stresses in the lateral ties became almost uniform across the cross-section. Alsayed et al. [84] also adopted a finite element model to predict the compressive behavior of CFRP-confined concrete in rectangular columns with section ellipticalization, but they failed to provide a practical design model.

Hadi et al. [49] experimentally studied the applicability of the section circularization for square hollow RC specimens under different loading conditions. Five groups of four hollow RC specimens (which had a cross-sectional width of 150 mm and a height of 800 mm) made from normal strength concrete were cast and tested. The specimens in the first group were RC hollow specimens, which served as reference specimens. The corners of the specimens in the second group were rounded to 20 mm and wrapped with a two-layer CFRP. The results showed that circularization increased the strength and ductility of the hollow column. Also, the CFRP wrap with fibers in the hoop direction mainly improved the performance of the specimens under concentric compression, while the CFRP wrap with fibers in the longitudinal direction mainly improved the performance of the specimens under eccentric compression.

Zeng et al. [85] presented a study on the compressive behavior of circularized square columns (CSCs) with FRP confinement, with the parameters of FRP wrapping schemes (including fully wrapped and partially wrapped), FRP volumetric ratio, sectional shapes and unconfined concrete strength being systematically investigated. Square columns with a width of 168 mm and their circularized counterparts were prepared and tensed under axial compressive loading. Circularization was achieved by casting concrete in the gap between existing columns and the circular formwork. The results of Zeng et al. [85] confirm that section circularization of square columns can significantly improve the effectiveness of FRP confinement and that strengthening square columns using section circularization in combination with partial FRP confinement is a promising and economical alternative to the full FRP strengthening technique. The combination of SC and the partial use of the FRP strengthening technique saved as much as 50% of the FRP material in the volumetric ratio, with strength and axial deformation capacities being comparable or even better than those of fully FRP-confined square columns.

Youssef et al. [86] investigated the effect of the section circularization of crumb rubber concrete with 0%, 10%, and 20% rubber contents. The circularization process was achieved by attaching four pre-cast concrete bolsters to the existing column, as was proposed in Hadi et al. [47]. One-layer and two-layer FRP wraps were used for the retrofitted columns. They reported that the axial stress–strain behavior of circularized square columns had a satisfactory performance and the crumb rubber concrete was able to exhibit a smoother transition zone than that of conventional concrete, especially for the case of using a 2-layer FRP wrap.

Mai et al. [87] presented the results of an experimental investigation on square and circularized square RC columns intermittently wrapped with CFRP jackets under different loading conditions. Twelve RC specimens consisting of eight square RC specimens with a 150-mm × 150-mm cross-section and an 800-mm height and four circularized square RC columns with a 212-mm diameter and an 800-mm height were tested under a concentric axial load, eccentric axial load and four-point flexural load. The test results showed that intermittent wrapping increased the strength and ductility of square RC columns. The test results also showed that circularization combined with intermittent wrapping significantly improved the strength and ductility of square RC specimens. The experimental axial load-bending moment interaction diagram showed that the best performance was achieved by intermittently CFRP wrapped circularized square RC specimens.

Table 1 shows the main thematic results for FRP-confined concrete columns with SM.

## 4. Section Curvilinearization

A small number of experimental studies [53,54,55,56,57,58,59,60,61] have been carried out on FRP-confined CRCs and CSCs. Pan et al. [52] studied the compressive behavior of FRP-confined CRCs, and the column slenderness effect of six FRP-confined CRCs with a single rise-to-span ratio (abbreviated as r/s ratio hereafter; see Figure 3 for the definitions of the rise and span of a CRC section) was investigated. It was found that the strengthening effect decreased with an increase in the slenderness ratio. The load carrying capacity of FRP-wrapped columns is 20% higher than that of ordinary reinforced concrete column when the slenderness ratio is less than 17.5. Jin et al. [54] and Lai et al. [56] both carried out axial compression tests on FRP-confined CSCs with only one r/s ratio. Lai et al. [56] performed an initial investigation in which only columns with a r/s ratio of 1/20 were examined. Test results from Lai et al. [56] also show that a CSC exhibits better axial stress–strain behavior than a square column with corner rounding. 

Zhao [58] examined the effect of the r/s ratio by testing eight FRP-confined CSCs covering three r/s ratios (i.e., 1/10, 1/15 and 1/20). All the CSCs tested by Zhao [58] were wrapped with four layers of CFRP and had a section width of 150 mm. The results showed that the maximum gain in compressive strength was 124% for the specimens with the highest r/s ratio (i.e., 1/10), and the minimum enhancement was 88% for the specimen with an r/s ratio of 1/20. More recently, Zhu et al. [59] carried out a systematic experimental study involving axial compression tests on FRP-confined CSCs including large-scale CSCs (sectional width = 300 mm) and covering four r/s ratios (1/7.5, 1/10, 1/15 and 1/20). Sixteen small-scale and ten large-scale FRP-confined square concrete columns with or without SC were tested under axial compression. A comparison of the results for columns of the two different sizes indicates that the effect of size is very limited in these FRP-confined CSCs. It was found that the compressive strength of FRP-confined concrete in CSCs can be effectively enhanced by using the SC method; however, the ultimate axial strain was not greatly affected.

**Figure 14:**
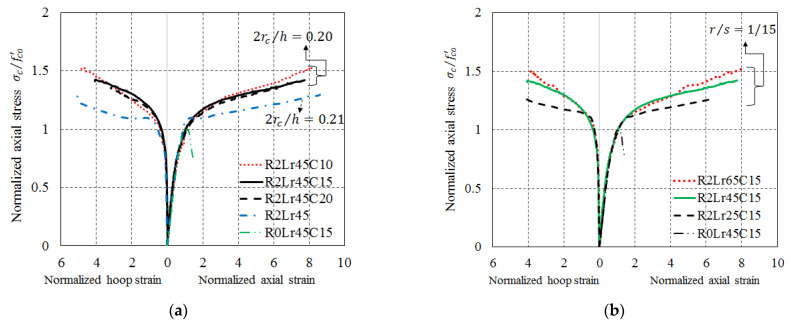
Normalized stress–strain curves of concrete in large-scale CRCs (Zeng et al. [60]). (**a**) CRCs with different *r*/*s* ratios. (**b**) CRCs with different corner radii.

Zeng et al. [60] reported that the SC technique substantially increases the effectiveness of FRP confinement in large-scale rectangular RC columns (with a sectional length of 450 mm and a sectional width of 300 mm). It was found that FRP-confined RC columns failed due to the abrupt rupture of the FRP jacket at or near one of the rounded corners near the column’s mid-height, and upon the removal of the ruptured FRP jacket and the spalled concrete, the exposed longitudinal steel bars were found to have buckled. The slope of the linear second segment of the stress–strain curve of FRP-confined concrete in a CRC was, in particular, much larger than that of the corresponding rectangular column without SC. Besides, the ultimate axial stress of FRP-confined concrete in CRCs increased with an increase in the r/s ratio and the corner radius ratio ($2r_{c}/h)$. Compared with a corresponding rectangular column, the CRCs with r/s ratios of 1/20, 1/15 and 1/10 achieved enhancements of 20%, 40% and 73% in ultimate axial stress, respectively (see Figure 14). Also, it was found that an r/s ratio of 1/15 and a corner radius ratio of 0.2 may be the optimum values for satisfactory enhancement in both FRP confinement effectiveness and ultimate axial stress without a large increase in the cross-sectional area for a rectangular RC column. Additionally, the FRP hoop strains at the centers of the rounded corners were generally smaller than those at the mid-width locations of side surfaces at the ultimate condition, and implementation of SC led to a more uniform FRP hoop strain distribution around the circumference of a rectangular column section.

Zeng [61] examined the responses of CFRP-confined large-scale CRCs subjected to eccentric compression. Twelve specimens were tested to investigate the effects of the rise-to-span ratio, load eccentricity, slenderness ratio and corner radius. It is shown that the ultimate axial strain at the extreme compression fiber (ECF) of an eccentrically-loaded CRC was larger than the ultimate axial strain in the corresponding concentrically-loaded CRC due to the strain gradient effect. An increase in the load eccentricity led to an increase in the maximum FRP hoop strain at the ECF at the ultimate condition, while the ultimate axial strain was independent of the load eccentricity. Also, an increase in column slenderness led to a decrease in the axial load-carrying capacity but an increase in the ultimate mid-height lateral displacement. In addition, an increase in column slenderness led to a decrease in the maximum FRP hoop strain at the ECF at the ultimate condition. The direct use of a concentric-loading stress–strain model for FRP-confined concrete in CRCs in a theoretical column model could provide reasonably satisfactory predictions for testing eccentrically-loaded CRCs, although the theoretical model underestimates the ductility of the test CRCs in particular, which is believed to be caused by the ignorance of strain gradient effects in the model. The design equations of GB 50608 [35] provide close predictions for the load-carrying capacity of the test CRCs. The theoretical column model generally predicts a slightly lower axial load at a given deformation for the eccentrically-loaded FRP-confined CRCs, which implies that the effect of the size of these columns may be insignificant, although more research needs to be conducted to clarify the effect of the size of these columns in the future.

## 5. Existing Models for FRP-Confined Concrete in Columns with SM

### 5.1. Model of Yan and Pantelides (2006)

Yan and Pantelides [88] proposed a confinement model which is applicable to FRP confined concrete with different cross-sectional geometries (circular, square and rectangular) and bond types (bonded FRP jacket or post-tensioned FRP shell). The Popovics model (Equation (1)) was applied to describe hardening behavior, and the Popovics and Saenz (Equation (2)) models were applied to describe the softening behavior of FRP-confined concrete compression members. 

The stress–strain curves for FRP-confined concrete in columns with SM are depicted by the following equations:

(1) $$\sigma_{c}=\{\begin{array}{cc} \frac{E_{c}\varepsilon_{c}}{1+\left(K-1\right)\cdot {\left(\varepsilon_{c}/\varepsilon_{cc}^{\prime }\right)}^{r}} & Hardening\\ f_{cc}^{\prime }\frac{K\cdot \left(\varepsilon_{c}/\varepsilon_{cc}^{\prime }\right)}{1+A\cdot \left(\varepsilon_{c}/\varepsilon_{cc}^{\prime }\right)+B\cdot {\left(\varepsilon_{c}/\varepsilon_{cc}^{\prime }\right)}^{2}+C\cdot {\left(\varepsilon_{c}/\varepsilon_{cc}^{\prime }\right)}^{3}} & Softening \end{array}$$

where $\sigma_{c}$ and $\varepsilon_{c}$ are axial stress and axial strain, $E_{c}$ is the elastic modulus of concrete, $f_{cc}^{\prime }$ is the ultimate axial stress of confined concrete and $\varepsilon_{cc}^{\prime }$ is the axial strain at peak stress. The details of the parameters, including r, K, A, B and C, in this model can be seen in Yan and Pantelides [88]. The ultimate axial stress $f_{cc}^{\prime }$, axial strain at peak stress $\varepsilon_{cc}^{\prime }$ and ultimate axial strain of confined concrete $\varepsilon_{cu}^{\prime }$, as proposed by Yan and Pantelides [88], are estimated by following equations:

(2) $$f_{cc}^{\prime }=\{\begin{array}{cc} f_{co}^{\prime }\left(\frac{-4.322+4.721\sqrt{1+4.193f_{l,e}/f_{co}^{\prime }}-2f_{l,e}/f_{co}^{\prime }}{0.0768\ln\left(f_{l,e}/f_{co}^{\prime }\right)+1.122}\right) & \left(f_{l,e}/f_{co}^{\prime }>0.2\right)\\ f_{co}^{\prime } & \left(f_{l,e}/f_{co}^{\prime }<0.2\right) \end{array}$$

(3) $$\varepsilon_{cc}^{\prime }=\{\begin{array}{cc} 6\varepsilon_{co}^{\prime }\left(f_{cc}^{\prime }/f_{co}^{\prime }-0.8\right) & \left(Hardening\right)\\ f_{cc}^{\prime }/E_{c}-\beta f_{cc}^{\prime } & \left(Softening\right) \end{array}$$

(4) $$\varepsilon_{cu}^{\prime }=f_{cu}^{\prime }\left(1+2\beta k_{\varepsilon }\varepsilon_{fu}\right)/E_{c}\;\;\;\;\;\;\;\;\;\;\left(Softening\right)$$

where $k_{\varepsilon }$ and $\varepsilon_{fu}$ are the FRP strain efficiency factor and the ultimate tensile strain of FRP; β is the relationship factor between the normalizing constant and the effective confinement ratio; $\varepsilon_{co}^{\prime }$ is the ultimate axial strain of unconfined concrete; $f_{co}^{\prime }$ is the strength of unconfined concrete and $f_{l,e}$ is the effective confining stress. The verification of the model was conducted by comparing test results and the predicted stress–strain curve of FRP-confined concrete in medium- and large-scale columns with bonded FRP jackets or post-tensioned FRP shells. It should be noted that many existing models are applicable to columns with section circularization/ellipticalization. Given that the strength of additional concrete/cement mortar is greater than that of the old concrete, the design of columns with SM can be conducted based on the strength of the old concrete, and therefore the design result can be conservative and acceptable for engineers.

### 5.2. Model of Zeng et al. (2017)

Zeng et al. [85] proposed a stress–strain model for FRP-confined concrete in square columns with section circularization. In the model, the stress–strain curve is depicted by the following equations from Lam and Teng [89], which consist of a parabolic first segment followed by a linear second segment:

(5) $$\sigma_{c}=\{\begin{array}{cc} E_{c}\varepsilon_{c}-\frac{{\left(E_{c}-E_{2}\right)}^{2}}{4f_{co}^{\prime }}\varepsilon_{c}^{2} & \left(0<\varepsilon_{c}<\varepsilon_{t}\right)\\ f_{co}^{\prime }+E_{2}\varepsilon_{c} & \left(\varepsilon_{t}\leq \varepsilon_{c}\leq \varepsilon_{cu}\right) \end{array}$$

where $\varepsilon_{t}$ is the transition axial strain between the two segments and $E_{2}$ is the second-segment slope. $E_{2}$ and $\varepsilon_{t}$ are defined by the following equations, respectively:

(6) $$E_{2}=\frac{f_{cc}^{\prime }-f_{co}^{\prime }}{\varepsilon_{cu}}$$

(7) $$\varepsilon_{t}=\frac{2f_{co}^{\prime }}{\left(E_{c}-E_{2}\right)}$$

**Figure 15:**
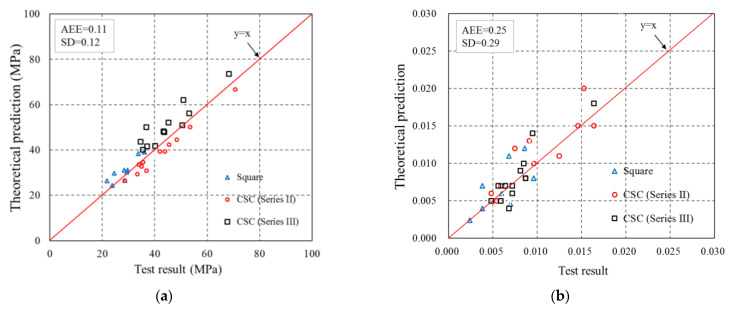
Comparisons of ultimate axial stresses and ultimate axial strains between test results and theoretical predictions (Zeng et al. [85]). (**a**) Ultimate axial stress. (**b**) Ultimate axial strain.

In Lam and Teng’s [89] model, the ultimate axial strain and the ultimate axial stress are evaluated as follows:

(8) $$\frac{f_{cc}^{\prime }}{f_{co}^{\prime }}=1+3.3\frac{f_{l,e}}{f_{co}^{\prime }}\;\frac{\varepsilon_{\mathrm{cu}}}{\varepsilon_{\mathrm{co}}}=1.75+12\frac{f_{l,e}}{f_{co}^{\prime }}{\left(\frac{\varepsilon_{h,rup}}{\varepsilon_{co}}\right)}^{0.45}$$

in which $\varepsilon_{h,rup}$ is the FRP hoop rupture strain. The vertical efficiency coefficient [33] for the partially FRP-confined concrete is used in this model. The comparisons presented in Zeng et al. [85] demonstrated that the combined use of the vertical confinement effectiveness coefficient provided by the design codes [33] and Lam and Teng’s [89] model can provide accurate predictions for both the ultimate axial stress and the ultimate axial strain of the partially FRP-confined concrete, as can be seen in Figure 15.

### 5.3. Model of GB-50608 (2010) for CRCs

As mentioned in the introductory section, one stress–strain model for FRP-confined concrete in CRCs is found in the Chinese national standard [35]. In the model, the stress–strain curve is depicted by the equation from Lam and Teng [89], as has been mentioned earlier. This model was established on the basis of results from small-scale FRP-confined CSCs with a single r/s ratio tested by Lai et al. [56].

The ultimate axial stress $f_{cc}^{\prime }$ and the ultimate axial strain $\varepsilon_{cu}$ are given by:

(9) $$f_{cc}^{\prime }=f_{co}^{\prime }+3\frac{E_{f}t_{f}}{R}\left(1-\frac{12.7}{\beta_{j}}\right)\varepsilon_{h,rup}$$

(10) $$\varepsilon_{cu}=0.0033+0.45\beta_{j}{}^{0.8}\varepsilon_{h,rup}{}^{1.45}$$

where $\varepsilon_{h,rup}$ is the FRP hoop rupture strain, $E_{f}$ is the elastic modulus of FRP jacket, $t_{f}$ is the thickness of the FRP jacket, $\beta_{j}$ is the confinement stiffness coefficient and R is the radius of the equivalent circular section. $\beta_{j}$ and R are defined by the following two equations, respectively:

(11) $$\beta_{j}=\frac{E_{f}\cdot t_{f}}{f_{co}^{\prime }R}$$

(12) R=(b+h)/π

where b and h are the section width and cross section height of the original column, respectively. The Chinese national standard specifies that the FRP rupture strain $\varepsilon_{h,rup}$ should be obtained from accompanying compression tests on 150 mm diameter circular concrete cylinders confined with a jacket of the same FRP and a practically reasonable confinement stiffness ratio. If such cylinder compression tests are not available, the hoop rupture strain $\varepsilon_{h,rup}$ can be conservatively taken as 0.5 times the rupture strain obtained from flat coupon tensile tests (i.e., $0.5\varepsilon_{f}$) for CFRP jackets. 

The revised version of the Chinese national standard (GB 50608 2020) specifies a new ultimate axial stress equation for FRP-confined concrete in CRCs based on studies presented in Zeng [61]:

(13) $$f_{cc}^{\prime }=f_{co}^{\prime }+3.5\frac{E_{f}t_{f}}{R}\left(k_{s,\sigma }-\frac{6.5}{\beta_{j}}\right)\varepsilon_{h,rup}$$

(14) $$k_{s,\sigma }=\left(1.25\frac{r_{c}}{b}+\frac{r_{c}}{h}+0.33\right)\cdot {\left(\frac{b}{h}\right)}^{0.4}\cdot \left(1+2.5r_{s}^{0.5}\right)$$

where $r_{c}$ is of the corner radius, $r_{s}$ is of the r/s ratio and $k_{s,\sigma }$ is the shape factor for ultimate axial stress.

### 5.4. Model of Lai et al. (2004) for CRCs

Lai et al. [56] proposed their stress–strain model for FRP-confined concrete in CSCs based on their own test data. This model is applicable to FRP-confined concrete in CSCs with an r/s ratio equal to or larger than 1/20. The stress_strain model consists of a nonlinear first segment and a linear second segment, which is expressed by the following equation:

(15) $$\sigma_{c}=\{\begin{array}{cc} \frac{\varepsilon_{c}}{M+M\varepsilon_{c}+Z\varepsilon_{c}^{2}} & \left(0\leq \varepsilon_{c}\leq \varepsilon_{t}\right)\\ \sigma_{cb}+E_{2}\left(\varepsilon_{c}-\varepsilon_{cb}\right) & \left(\varepsilon_{t}\leq \varepsilon_{c}\leq \varepsilon_{cu}\right) \end{array}$$

where $\sigma_{cb}$ and $\varepsilon_{cb}$ are the axial stress and axial strain at the transition point between the two segments. The constants M, N and Z are determined by three boundary conditions: (1) the initial slope of the stress–strain curve is equal to the elastic modulus of unconfined concrete $E_{c}$, (2) the curve passes through the transition point ($\varepsilon_{cb},\sigma_{cb}$) and (3) the slope of the stress–strain curve at the transition point is equal to the slope of the linear second segment ($E_{2}$) to ensure a smooth transition between the two segments. The three constants are calculated by the following equations for the stress–strain curve with an ascending and a descending second segment, respectively (Lat et al. [56]):

(16) $$M=\frac{1}{E_{c}},\;N=\left[\frac{1}{E_{p}}-\frac{2}{E_{c}}+\frac{1}{E_{p}}\frac{E_{2}}{E_{p}}\right]\frac{1}{\varepsilon_{cb}},\;Z=\left[\frac{1}{E_{c}}-\frac{1}{E_{p}}\frac{E_{2}}{E_{p}}\right]\frac{1}{\varepsilon_{cb}^{2}}$$

where $E_{p}=\sigma_{cb}/\varepsilon_{cb}$ is the secant modulus of concrete.

The slope of the linear second segment $E_{2}$ is calculated using the following equations, which were developed based mainly on the interpretation of their own test data:

(17) $$E_{2}/E_{c}=k_{2}ln\left(\beta_{j}\right)-k_{3}\;\;\;\;\;\;\;\;\;\;\;\;\;\left(\beta_{j}>\beta_{j0}\right)$$

(18) $$E_{2}/E_{c}=k_{4}ln\left(\beta_{j}\right)-k_{5}\;\;\;\;\;\;\;\;\;\;\;\;\;\left(\beta_{j}<\beta_{j0}\right)$$

where $k_{2}$, $k_{3}$, $k_{4}$ and $k_{5}$ are constants determined using regression analysis, $\beta_{j}$ is the confinement stiffness ratio (Equation (11)) and R is the radius of the equivalent circular section of the curvilinearized rectangular section (Equation (12)).

The predictive equations for the transition axial strain and stress are given by:

(19) $$\frac{\varepsilon_{cb}}{\varepsilon_{co}}=1+0.0110\beta_{j}$$

(20) $$\frac{\sigma_{cb}}{f_{co}^{\prime }}=1+0.0568\beta_{j}{}^{0.46}$$

The ultimate axial strain and ultimate axial stress are calculated by:

(21) $$\varepsilon_{cc}=\varepsilon_{co}\left(1+178.9\beta_{j}^{0.75}\varepsilon_{h,rup}^{1.25}\right)$$

(22) $$f_{cc}^{\prime }=f_{co}^{\prime }\left(1+3.366\beta_{j}\varepsilon_{h,rup}\right)$$

Lai et al. [56] suggested that a reduction coefficient of 0.91 be used for $\varepsilon_{h,rup}$ (i.e., $\varepsilon_{h,rup}=0.91\varepsilon_{f}$), but they provided no experimental or theoretical basis for this coefficient. It should be noted that the model of Lai et al. [56] does not include the r/s ratio of curved sides as a parameter.

**Figure 16:**
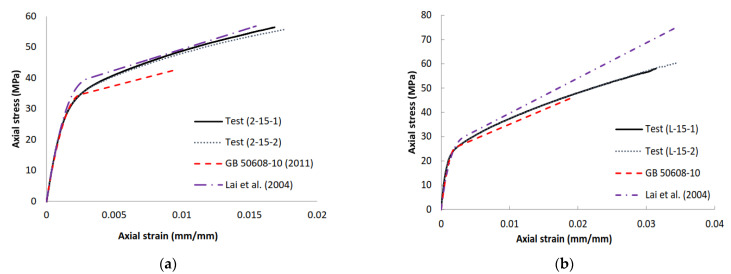
Comparison of stress–strain curves between the existing design-oriented stress–strain models and the test results of CSCs (Zhu [57]). (**a**) Small-scale specimens. (**b**) Large-scale specimens.

Zhu et al. [59] reported that the stress–strain model in the Chinese national standard [35] significantly underestimates the ultimate axial stresses and ultimate axial strains of FRP-confined concrete in the test CSCs (Figure 16). Figure 16 shows the stress–strain curves predicted with GB-50608 [35] in comparison with the test curves for the four FRP-confined CSCs from Zhu et al. [59]. It is evident that the predicted stress–strain curves are far shorter than the test curves, and thus both the ultimate axial stresses and ultimate axial strains are greatly underestimated. The predicted second segment slopes are, however, reasonably close to the corresponding experimental slopes. The inaccuracy of this model is mainly due to the limited test data based on which this model was derived; these test data were all small-scale FRP-confined CSCs with a single r/s ratio from Lai et al. [56]. It can be seen from Figure 16 that the model of Lai et al. [56] predicts the stress–strain curves of small-scale specimens very well in terms of the ultimate condition and the second-stage slope of the stress–strain curve; however, it significantly overestimates the response of the large-scale specimens, as seen in Figure 16. Zeng et al. [60] also reported that the stress–strain model in the Chinese national standard (GB-50608 [35]) significantly underestimates the ultimate axial stresses and ultimate axial strains of FRP-confined concrete in the test CRCs. As a result, a more accurate stress–strain model for FRP-confined concrete in CRCs needs to be developed. Nevertheless, the revised model given in GB-50608 2020 needs to be assessed against a database with extensive test results.

The axial load-carrying capacity and the corresponding section moment capacity of an eccentrically-loaded FRP-confined rectangular RC column are calculated by (GB 50608 [35]):

(23) $$\{\begin{array}{c} N\leq \alpha_{1}f_{cc}^{\prime }bx+\sigma_{s}^{\prime }A_{s}^{\prime }-\sigma_{s}A_{s}\\ Ne_{max}\leq \alpha_{1}f_{cc}^{\prime }bx\left(\frac{h}{2}-\frac{x}{2}\right)+\sigma_{s}^{\prime }A_{s}^{\prime }\left(\frac{h}{2}-a_{s}^{\prime }\right)+\sigma_{s}A_{s}\left(\frac{h}{2}-a_{s}\right) \end{array}$$

(24) $$e_{max}=max\left(\eta e_{i},e_{2}+e_{a}\right)$$

(25) $$e_{i}=e_{0}+e_{a}$$

(26) $$e_{0}=0.6e_{2}+0.4e_{1}\leq 0.4e_{2}$$

where h is the dimension of the cross-section in the direction of eccentric loading; b is the dimension of the cross-section in the direction perpendicular to the eccentric loading direction; x is the depth of the compression zone; $A_{s}^{\prime }$ and $\sigma_{s}^{\prime }$ are the total area and stress of the longitudinal steel reinforcement at the compression side or more compressive side; $A_{s}$ and $\sigma_{s}$ are the total area and stress of the longitudinal steel reinforcement at the tension side or less compressive side; $a_{s}^{\prime }$ is the distance between the point of the resultant force of the longitudinal steel reinforcement at the more compressive side and the extreme compression edge; $a_{s}$ is the distance between the point of the resultant force of the longitudinal steel reinforcement at the tension side or less compressive side and the extreme tension (or less compressive) edge; $e_{i}$ is the initial load eccentricity; $e_{0}$ is the equivalent load eccentricity; $e_{a}$ is the additional load eccentricity; η is the enhancement coefficient for the initial load eccentricity to account for the slenderness effect; and $e_{1}$ and $e_{2}$ are the load eccentricities at the two ends of the column, respectively. It should be noted that $e_{2}$ is designated as the one with a larger absolute value and is always assigned a non-negative value. This means that $e_{1}$ has a negative value when the column is bent in double curvature. The theoretical model was revised for FRP-confined CRCs by Zeng [61].

Eccentric compression tests on a series of large-scale long FRP-confined CRCs with different slenderness ratios and load eccentricities have been conducted. The effects of load eccentricity, the edge rise-to-span ratio and the slenderness ratio have been investigated. The test results were compared with the numerical results from predictions of the design equations in GB 50608 [35], as given above. It was found that the design equations of GB 50608 [35] provide close predictions for the load-carrying capacity of the test CRCs (Zeng [61]).

In Hadi et al. [47], an axial load-bending moment (P–M) interaction diagram with a continuous curve was used to determine the axial load (P) and bending moment (M) of a given RC column cross section. Each point on this curve includes two components, namely, the value of the axial loading and the corresponding bending moment. In the study of Hadi et al. [47], an experimental interaction diagram was drawn based on four points: (1) a pure axial load of the column under a concentric load, (2) two points of eccentric loads of 15 and 25 mm in which the axial loads were recorded from the testing machine and the bending moments were calculated. In order to draw a theoretical interaction diagram (P-M), a confined concrete model of Lam and Teng [89] was utilized. The accuracy of this procedure has been verified by Hadi et al. [47].

## 6. Concluding Remarks and Future Study

This paper has presented a comprehensive review on FRP-confined non-circular columns with SM before FRP jacketing. The SM approach generally includes modifying a square section into a circular one, modifying a rectangular section into an elliptical/oval one and modifying a square/rectangular section into a curvilinearized square/rectangular section. The effects of key parameters on the effectiveness of FRP confinement are discussed, and different methods of implementing SM in real applications is briefly introduced. The findings of the review further confirm the effectiveness of the SM approach. Additionally, existing theoretical models for FRP-confined concrete in columns with SM are summarized. Further research opportunities associated with FRP-confined non-circular columns with SM are identified. The following conclusions can be drawn:

(1) Extensive studies have conducted been on FRP-confined concrete columns with section circularization/ellipticalization, leading to a unified conclusion that the effectiveness of FRP confinement is substantially enhanced owning to section circularization/ellipticalization.
(2) Existing models for FRP-confined concrete in columns with SM are mainly conservative, and they are basically applicable to columns with section circularization/ellipticalization. Design results can be conservative and acceptable for engineers based on the strength of the old concrete, given that the strength of the additional concrete/cement mortar is greater than the old concrete.
(3) Adding new concrete bolsters and casting new concrete in FRP stay-in-place formworks are both applicable in the context of the SM of columns before FRP jacketing. However, the difference of the above SM approaches and the effects of the above SM approaches on FRP confinement mechanism remain unclear.
(4) An increase of the cross-sectional area of columns as a result of SC is much smaller than that of columns with section circularization/ellipticalization, and implementing SC to rectangular columns before FRP jacketing also substantially enhances the effectiveness of FRP confinement.
(5) Studies on FRP-confined concrete in curvilinearized square/rectangular columns are far from adequate when it comes to establishing an accurate stress–strain model for FRP-confined concrete in curvilinearized square/rectangular columns.

Future studies are required for FRP-confined concrete columns with SM. The effects of SM approaches on FRP confinement mechanism, particularly the approach that is associated with 3D printing additional concrete/cement mortar bolsters, need to be explored. Moreover, the behavior of concrete columns with SM using high-performance cementitious material bolsters needs to be understood thoroughly. Additionally, there is a lack of studies on the seismic behavior of FRP-confined concrete columns with SM. Also, novel forms structural members with a curvilinearized square/rectangular section need to be developed and investigated.

## Figures and Tables

**Table 1:** Main thematic results for FRP-confined concrete columns with SM.

References	SCir	SEll	SCurv	Main Thematic Results
Priestley and Seible [40]		√		Confinement efficiency for columns with corner rounding was only 50% of that for columns with SCir.
Teng and Lam [43]		√		The confinement effectiveness decreased with the a/b ratio and the stress–strain curve exhibited a descending branch if the effective confinement ratio was equal to or less than 0.11. A strength model was developed for FRP-confined concrete in elliptical columns.
Yan et al. [81]	√	√		SCir/SEll yielded the post-peak hardening behavior of FRP-confined square/rectangular columns. A higher axial compressive strength and higher energy absorption were observed for SM square/rectangular columns with post-tensioned FRP shells compared with members confined using bonded FRP jackets.
Parvin and Schroeder [82]		√		The effectiveness of CFRP wrapping was substantially reduced for eccentric loading compared with concentric loading, and the CFRP jacket was more effective in the axial direction than the CFRP jacket in the hoop direction for eccentrically loaded columns.
Hadi et al. [47]	√			Both corner rounding and section circularization were effective is enhancing the compressive behavior of FRP-confined concrete in square columns. The added concrete covers effectively enhanced the load carrying capacity of the column by increasing the cross-sectional area and increasing the effectiveness of the FRP confinement.
Pham et al. [48]	√			Section circularization is effective in enhancing the FRP confinement efficiency. The concrete covers with a higher strength exhibited a higher load-carrying capacity than the concrete covers with a lower strength.
Hadi and Tran [83]	√			The performances of the original columns were improved significantly after being strengthened using SCir. The columns with SCir with increased FRP thickness helped to relocate the failure of the beam-column connection from the joint location to any preferred location in the beam span.
Alsayed et al. [84]		√		CFRP confinement increased both the strength and ductility of confined concrete in rectangular RC columns with SE. The stresses in the lateral ties became almost uniform across the cross-section owning to the confinement provided by the CFRP wrap.
Zeng et al. [85]	√			SCir can significantly improve the effectiveness of FRP confinement. The combination of SCir and the partial use of the FRP strengthening technique saved as much as 50% of the FRP material in the volumetric ratio.
Youssef et al. [86]	√			The axial stress–strain behavior of circularized square columns had a satisfactory performance, and the crumb rubber concrete was able to exhibit a smoother transition zone than that of conventional concrete.
Mai et al. [87]	√			SCir combined with intermittent wrapping significantly improved the strength and ductility of square RC columns.
Pan et al. [52]			√	The strengthening effect decreased with an increase in the slenderness ratio. The load carrying capacity of FRP-wrapped columns was 20% higher than that of an ordinary reinforced concrete column when the slenderness ratio was less than 17.5.
Lai et al. [56]			√	A CSC exhibits better axial stress–strain behavior than a square column with corner rounding.
Zhao [58]			√	The maximum gain in compressive strength was 124% for the specimens with a highest r/s ratio (i.e., 1/10), and the minimum enhancement was 88% for the specimen with an r/s ratio of 1/20.
Zhu et al. [59]			√	The size effect is very limited in these FRP-confined CSCs. The compressive strength of FRP-confined concrete in CSCs can be effectively enhanced by using the SC method, but the ultimate axial strain was not greatly affected.
Zeng et al. [60]			√	The slope of the linear second segment of the stress–strain curve of FRP-confined concrete in a CRC was much larger than that of the corresponding rectangular column without SCurv. Besides, the ultimate axial stress of FRP-confined concrete in CRCs increased with an increase in the r/s ratio and the corner radius ratio. Compared with a corresponding rectangular column, the CRCs with r/s ratios of 1/20, 1/15, and 1/10 achieved enhancements of 20%, 40%, and 73%, respectively, in ultimate axial stress. Also, an r/s ratio of 1/15 and a corner radius ratio of 0.2 may be the optimum values for satisfactory enhancement in terms of both FRP confinement effectiveness and ultimate axial stress.

Note: SCir—section circularization; SEll—section ellipticalization; SCurv—section curvilinearization; √—applicable.

## Data Availability

Some or all data, models, or code that support the findings of this study are available from the corresponding author upon reasonable request.

## References

[1] Teng J.G., Chen J.F., Smith S.T., Lam L. (2002). FRP-Strengthened RC Structures.

[2] Abokwiek R., Abdalla J.A., Hawileh R.A., El Maaddawy T. (2021). RC Columns Strengthened with NSM-CFRP Strips and CFRP Wraps under Axial and Uniaxial Bending: Experimental Investigation and Capacity Models. J. Compos. Constr..

[3] Wu Y.-F., Jiang C. (2013). Effect of load eccentricity on the stress–strain relationship of FRP-confined concrete columns. Compos. Struct..

[4] Shayanfar J., Barros J.A.O., Rezazadeh N. (2021). Generalized Analysis-oriented model of FRP confined concrete circular columns. Compos. Struct..

[5] Lai M.H., Liang Y.W., Wang Q., Ren F.M., Chen M.T., Ho J.C.M. (2020). A stress-path dependent stress-strain model for FRP-confined concrete. Eng. Struct..

[6] Koutas L.N., Tetta Z., Bournas D.A., Triantafillou T.C. (2019). Strengthening of concrete structures with textile reinforced mortars: State-of-the-art review. J. Compos. Constr..

[7] Al Ajarmeh O.S., Manalo A.C., Benmokrane B., Karunasena K., Ferdous W., Mendis P. (2020). Hollow concrete columns: Review of structural behavior and new designs using GFRP reinforcement. Eng. Struct..

[8] Bai Y.-L., Yan Z.-W., Ozbakkaloglu T., Gao W.-Y., Zeng J.-J. (2021). Mechanical behavior of large-rupture-strain (LRS) polyethylene naphthalene fiber bundles at different strain rates and temperatures. Constr. Build. Mater..

[9] Song J., Gao W.-Y., Ouyang L.-J., Zeng J.-J., Yang J., Liu W.-D. (2021). Compressive behavior of heat-damaged square concrete prisms confined with basalt fiber-reinforced polymer jackets. Eng. Struct..

[10] Liao J.-J., Zeng J.-J., Gong Q.-M., Quach W.-M., Gao W.-Y., Zhang L.-H. (2022). Design-oriented stress-strain model for FRP-confined ultra-high performance concrete (UHPC). Constr. Build. Mater..

[11] Zhou J.-K., Lin W.-K., Guo S.-X., Zeng J.-J., Bai Y.-L. (2022). Behavior of FRP-confined FRP spiral reinforced concrete square columns (FCFRCs) under axial compression. J. Build. Eng..

[12] Ferrotto M.F., Fischer O., Cavaleri L. (2018). A strategy for the finite element modeling of FRP-confined concrete columns subjected to preload. Eng. Struct..

[13] Bai Y.-L., Zhang Y.-F., Jia J.-F., Mei S.-J., Han Q., Dai J.-G. (2021). Simplified plasticity damage model for large rupture strain (LRS) FRP-confined concrete. Compos. Struct..

[14] Pellegrino C., Modena C. (2011). Analytical model for FRP confinement of concrete columns with and without internal steel reinforcement. J. Compos. Constr..

[15] Zeng J.-J., Chen S.-P., Zhuge Y., Gao W.-Y., Duan Z.-J., Guo Y.-C. (2021). Three-dimensional finite element modeling and theoretical analysis of concrete confined with FRP rings. Eng. Struct..

[16] Zeng J.-J., Duan Z.-J., Gao W.Y., Bai Y.-L., Ouyang L.-J. (2020). Compressive behavior of FRP-wrapped seawater sea-sand concrete with a square cross-section. Constr. Build. Mater..

[17] Liao J.J., Yang K.Y., Zeng J.J., Quach W.M., Ye Y.Y., Zhang L. (2021). Compressive behavior of FRP-confined ultra-high performance concrete (UHPC) in circular columns. Eng. Struct..

[18] Guo Y.-C., Xiao S.-H., Shi S.-H., Zeng J.-J., Wang W.-Q., Zhao H.-C. (2020). Axial compressive behavior of concrete-filled FRP-steel wire reinforced thermoplastics pipe hybrid columns. Compos. Struct..

[19] Fanaradelli T., Rousakis T., Karabinis A. (2019). Reinforced concrete columns of square and rectangular section, confined with FRP—Prediction of stress and strain at failure. Compos. Part B Eng..

[20] Isleem H.F., Wang D.Y., Wang Z.Y. (2018). Modeling the axial compressive stress-strain behaviour of CFRP-confined rectangular RC columns under monotonic and cyclic loading. Compos. Struct..

[21] Ilki A., Peker O., Karamuk E., Demir C., Kumbasar N. (2008). FRP retrofit of low and medium strength circular and rectangular reinforced concrete columns. J. Mater. Civ. Eng..

[22] Ozcan O., Binici B., Ozcebe G. (2010). Seismic strengthening of rectangular reinforced concrete columns using fiber reinforced polymers. Eng. Struct..

[23] Pessiki S., Harries K.A., Kestner J., Sause R., Ricles J.M. (2001). The axial behavior of concrete confined with fiber reinforced composite jackets. J. Compos. Constr..

[24] Yuan F., Wu Y.-F., Li C.-Q. (2017). Modelling plastic hinge of FRP-confined RC columns. Eng. Struct..

[25] Eid R., Paultre P. (2017). Compressive behavior of FRP-confined reinforced concrete columns. Eng. Struct..

[26] Chellapandian M., Prakash S.S., Rajagopal A. (2018). Analytical and finite element studies on hybrid FRP strengthened RC columns elements under axial and eccentric compression. Compos. Struct..

[27] Lam L., Teng J.G. (2003). Design-oriented stress-strain model for FRP-confined concrete in rectangular columns. J. Reinf. Plast. Compos..

[28] Rocca S., Galati N., Nanni A. (2008). Review of design guidelines for FRP confinement of reinforced concrete columns of noncircular cross sections. J. Compos. Constr..

[29] Wu Y.-F., Wei Y.-Y. (2010). Effect of cross-sectional aspect ratio on the strength of CFRP-confined rectangular concrete columns. Eng. Struct..

[30] Ren F.M., Liang Y.W., Ho J.C.M., Lai M.H. (2020). Behaviour of FRP tube-concrete-encased steel composite columns. Compos. Struct..

[31] Guo Y.C., Xiao S.H., Luo J.W., Ye Y.Y., Zeng J.J. (2018). Confined concrete in square columns partially wrapped with FRP strips: Axial compressive behavior and strain distributions by particle image velocimetry sensing technique. Sensors.

[32] (2017). Guide for the Design and Construction of Externally Bonded FRP Systems for Strengthening Concrete Structures.

[33] (2013). Guide for the Design and Construction of Externally Bonded FRP Systems for Strengthening Existing Structures.

[34] Concrete Society (2012). Design Guidance for Strengthening Concrete Structures using Fibre Composite Materials.

[35] (2010). Technical Code for Infrastructure Application of FRP Composites.

[36] Rocca S., Galati N., Nanni A. Large-size reinforced concrete columns strengthened with carbon FRP: Experimental evaluation. Proceedings of the 3rd International Conference on FRP Composites in Civil Engineering.

[37] Wang L.-M., Wu Y.-F. (2008). Effect of corner radius on the performance of CFRP-confined square concrete columns: Test. Eng. Struct..

[38] Seible F., Priestley M.J.N. Strengthening of rectangular bridge columns for increased ductility. Proceedings of the Symposium on Practical Solutions for Bridge Strengthening and Rehabilitation.

[39] Priestley M.J.N., Seible F., Xiao Y., Verma R. (1994). Steel jacket retrofitting of reinforced concrete bridge columns for enhanced shear strength—Part 1: Theoretical considerations and test design. ACI Struct. J..

[40] Priestley M.J.N., Seible F. (1995). Design of seismic retrofit measures for concrete and masonry structures. Constr. Build. Mater..

[41] Seible F., Priestley M.J.N., Hegemier G.A., Innamorato D. (1997). Seismic retrofit of RC columns with continuous carbon fiber jacket. J. Compos. Constr..

[42] Saadatmanesh H., Ehsani M.R., Jin L. (1997). Seismic retrofitting of rectangular bridge columns with composite straps. Earthq. Spectra.

[43] Teng J.G., Lam L. (2002). Compressive behavior of carbon fiber reinforced polymer-confined concrete in elliptical columns. J. Struct. Eng..

[44] Teng J.G., Wu J.Y., Casalboni S., Xiao Q.G., Zhao Y. (2016). Behavior and modeling of fiber-reinforced polymer-confined concrete in elliptical columns. Adv. Struct. Eng..

[45] El Maaddawy T., El Sayed M., Abdel-Magid B. (2010). The effects of cross-sectional shape and loading condition on performance of rein-forced concrete members confined with carbon fiber reinforced polymers. Mater. Des..

[46] Yan Z., Pantelides C.P. (2011). Concrete column shape modification with FRP shells and expansive cement concrete. Constr. Build. Mater..

[47] Hadi M.N.S., Pham T.M., Lei X. (2013). New method of strengthening reinforced concrete square columns by circularizing and wrap-ping with fiber-reinforced polymer or steel straps. J. Compos. Constr..

[48] Pham T.M., Doan L.V., Hadi M.N.S. (2013). Strengthening square reinforced concrete columns by circularisation and FRP confinement. Constr. Build. Mater..

[49] Hadi M.N.S., Jameel M.T., Sheikh M.N. (2017). Behavior of circularized hollow RC columns under different loading conditions. J. Compos. Constr..

[50] Pantelides C.P., Yan Z.H. (2007). Confinement model of concrete with externally bonded FRP jackets or posttensioned FRP shells. J. Struct. Eng..

[51] Parvin A., Wang W. (2001). Behavior of FRP jacketed concrete columns under eccentric loading. J. Compos. Constr..

[52] Pan J.L., Xu T., Hu Z.J. (2007). Experimental investigation of load carrying capacity of the slender reinforced concrete columns wrapped with FRP. Constr. Build. Mater..

[53] Jin X.N. (2002). Experimental Research on Mechanical Properties of Axisymmetric Confined Concrete. Ph.D. Thesis.

[54] Jin X.N., Pan J.L., Lai W.H., Wang Y.G. (2002). Mechanical behavior of short reinforced concrete columns wrapped with FRP under axial compression. Low Temp. Archit. Technol..

[55] Lai W.H. (2003). Experimental Research on Stress-Strain Behavior of FRP-Confined Concrete. Master’s Thesis.

[56] Lai W.H., Pan J.L., Jin X.N. (2004). Compressive stress-strain behavior of concrete confined by fiber reinforced polymer. Ind. Constr..

[57] Zhu J.Y. (2014). FRP-Confined Curvilinearized Square Concrete Columns under Axial Compression. Master’s Thesis.

[58] Zhao W. (2012). Experimental Behaviour of Curvilinearised Square Columns Confined with Fiber-Reinforced Polymer. Master’s Thesis.

[59] Zhu J.Y., Lin G., Teng J.G., Chan T.M., Zeng J.J., Li L.J. (2020). FRP-confined square concrete columns with section curvilinearization under axial compression. J. Compos. Constr..

[60] Zeng J.J., Lin G., Teng J.G., Li L.J. (2021). Axial compressive behavior of large-scale FRP-confined rectangular RC columns with section curvilinearization. J. Compos. Constr..

[61] Zeng J.J. (2017). Behaviour and Modelling of Large-Scale FRP-Confined Rectangular and Curvilinearized Rectangular RC Columns. Ph.D. Thesis.

[62] Teng J.-G., Wang Z., Yu T., Zhao Y., Li L.-J. (2018). Double-tube concrete columns with a high-strength internal steel tube: Concept and behaviour under compression. Adv. Struct. Eng..

[63] Guo Y.-C., Ye Y.-Y., Lin G., Lv J.-F., Bai Y.-L., Zeng J.-J. (2020). Effective usage of high strength steel tubes: Axial compressive behavior of hybrid FRP-concrete-steel solid columns. Thin-Walled Struct..

[64] Chen G.M., Lan Z.H., Xiong M.X., Xu Z. (2020). Compressive behavior of FRP-confined steel-reinforced high strength concrete columns. Eng. Struct..

[65] Ozbakkaloglu T. (2015). A novel FRP–dual-grade concrete-steel composite column system. Thin-Walled Struct..

[66] Fanggi B.A.L., Ozbakkaloglu T. (2015). Square FRP-HSC-steel composite columns: Behavior under axial compression. Eng. Struct..

[67] Ye Y.-Y., Liang S.-D., Feng P., Zeng J.-J. (2021). Recyclable LRS FRP composites for engineering structures: Current status and future opportunities. Compos. Part B Eng..

[68] Ouyang L.J., Chai M.X., Song J., Hu L.L., Gao W.Y. (2021). Repair of thermally damaged circular concrete cylinders with basalt fiber-reinforced polymer jackets. J. Build. Eng..

[69] Shi C., Wu Z., Xiao J., Wang D., Huang Z., Fang Z. (2015). A review on ultra high performance concrete: Part I. Raw materials and mixture design. Constr. Build. Mater..

[70] Dong Z., Wu G., Zhao X.-L., Zhu H., Shao X. (2019). Behaviors of hybrid beams composed of seawater sea-sand concrete (SWSSC) and a prefabricated UHPC shell reinforced with FRP bars. Constr. Build. Mater..

[71] Yu K.Q., Yu J.-T., Dai J.-G., Lu Z.-D., Shah S.P. (2018). Development of ultra-high performance engineered cementitious composites using polyethylene (PE) fibers. Constr. Build. Mater..

[72] Liu Y.W., Zhang Z.H., Shi C.J., Zhu D.J., Li N., Deng Y.L. (2020). Development of ultra-high performance geopolymer concrete (UHPGC): Influence of steel fiber on mechanical properties. Cem. Concr. Compos..

[73] Bertola N., Schittz P., Denaria E., Brühwiler E. (2021). A Review of the Use of UHPFRC in Bridge Rehabilitation and New Con-struction in Switzerland. Front. Built Environ..

[74] Huang B.-T., Yu J., Wu J.-Q., Dai J.-G., Leung C.K.Y. (2020). Seawater sea-sand Engineered Cementitious Composites (SS-ECC) for marine and coastal applications. Compos. Commun..

[75] Al-Gemeel A.N., Zhuge Y. (2019). Using textile reinforced engineered cementitious composite for concrete columns confinement. Compos. Struct..

[76] Jiang J.F., Jiang C., Li B.B., Feng P. (2019). Bond behavior of basalt textile meshes in ultra-high ductility cementitious composites. Compos. Part B Eng..

[77] Pan B.Z., Liu F., Zhuge Y., Zeng J.-J., Liao J.J. (2022). ECC/UHPFRCC with and without FRP reinforcement for structural strengthening/repairing: A state-of-the-art review. Constr. Build. Mater..

[78] D’Antino T., Focacci F., Sneed L.H., Pellegrino C. (2020). Shear strength model for RC beams with U-wrapped FRCM composites. J. Compos. Constr..

[79] Ye Y.Y., Smith S.T., Zeng J.J., Zhuge Y., Quach W.M. (2021). Novel Ultra-High-Performance Concrete Composite Plates Rein-forced with FRP Grid: Development and Mechanical Behaviour. Compos. Struct..

[80] Zheng Y.-Z., Wang W.-W., Mosalam K.M., Zhu Z.-F. (2018). Mechanical behavior of ultra-high toughness cementitious composite strengthened with fiber reinforced polymer grid. Compos. Struct..

[81] Yan Z., Pantelides C.P., Reaveley L.D. (2006). Fiber-reinforced polymer jacketed and shape-modified compression members: I—Experimental behavior. ACI Struct. J..

[82] Parvin A., Schroeder J.M. (2008). Investigation of eccentrically loaded CFRP-confined elliptical concrete columns. J. Compos. Constr..

[83] Hadi M.N.S., Tran T.M. (2014). Retrofitting nonseismically detailed exterior beam-column joints using concrete covers together with CFRP jacket. Constr. Build. Mater..

[84] Alsayed S.H., Almusallam T.H., Ibrahim S.M., Al-Hazmi N.M., Al-Salloum Y.A., Abbas H. (2014). Experimental and numerical investigation for compression response of CFRP strengthened shape modified wall-like RC column. Constr. Build. Mater..

[85] Zeng J.-J., Guo Y.-C., Gao W.-Y., Li J.-Z., Xie J.-H. (2017). Behavior of partially and fully FRP-confined circularized square columns under axial compression. Constr. Build. Mater..

[86] Youssf O., Hassanli R., Mills J. (2017). Retrofitting square columns using FRP-confined crumb rubber concrete to improve confinement efficiency. Constr. Build. Mater..

[87] Mai A.D., Sheikh M.N., Hadi M.N.S. (2019). Performance evaluation of intermittently CFRP wrapped square and circularised square reinforced concrete columns under different loading conditions. Struct. Infrastruct. Eng..

[88] Yan Z., Pantelides C.P. (2006). Fiber-Reinforced Polymer Jacketed and shape-Modified Compression Members: II—Model. ACI Struct. J..

[89] Lam L., Teng J.G. (2003). Design-oriented stress-strain model for FRP-confined concrete. Constr. Build. Mater..

